# Supplementary material for: Microgeographic Wing-Shape Variation in Aedes albopictus and Aedes scapularis (Diptera: Culicidae) Populations
Source: Insects. 2020 Dec 3;11(12):862. doi: 10.3390/insects11120862 (PMC7761735; doi:10.3390/insects11120862)
Supplement: Supplementary file 1 [file insects-11-00862-s001.pdf]

Table S1. Tukey pairwise comparisons in *Ae. scapularis* populations.

Parks	ANH	AFV	BLM	PQR	STD
**ANH**	-	0.4586	1	0.8598	0.05426
**AFV**	2.348	-	0.46667	0.06152	0.0001413
**BLM**	0.01803	2.33	-	0.854	0.05241
**PQR**	1.401	3.749	1.419	-	0.4293
**STD**	3.815	6.164	3.833	2.415	-

Table S2. Tukey pairwise comparisons in *Ae. albopictus* populations.

Parks	ANH	BLM	PQR	PRV	SHL
**ANH**	-	0.8747	1	0.1539	0.05165
**BLM**	1.35	-	0.8729	0.6641	0.3726
**PQR**	0.006199	1.356	-	0.1525	0.05107
**PRV**	3.251	1.902	3.258	-	0,.902
**SHL**	3.912	2.562	3.918	0.6606	-

Table S3. Procrustes distances (below diagonal) and *P* values (above diagonal) for *Ae. scapularis* populations.

Parks	ALV	ANH	BLM	PQR	STD
**ALV**	-	<0.0001	<0.0001	0.0075	0.0012
**ANH**	0.0248	-	0.502	0.0467	0.0096
**BLM**	0.0254	0.0093	-	0.0223	0.0024
**PQR**	0.0183	0.0145	0.0159	-	0.1904
**STD**	0.0201	0.0161	0.0182	0.0126	-

Table S4. Procrustes distances (below diagonal) and *p* values (above diagonal) for *Ae. albopictus* populations.

Parks	ANH	BLM	PQR	PRV	SHL
**ANH**	-	0.0384	0.1528	0.0395	0.5687
**BLM**	0.018	-	0.0812	0.4476	0.2782
**PQR**	0.0138	0.0145	-	0.0076	0.4245
**PRV**	0.0161	0.0111	0.0163	-	0.3332
**SHL**	0.0121	0.014	0.0115	0.012	-

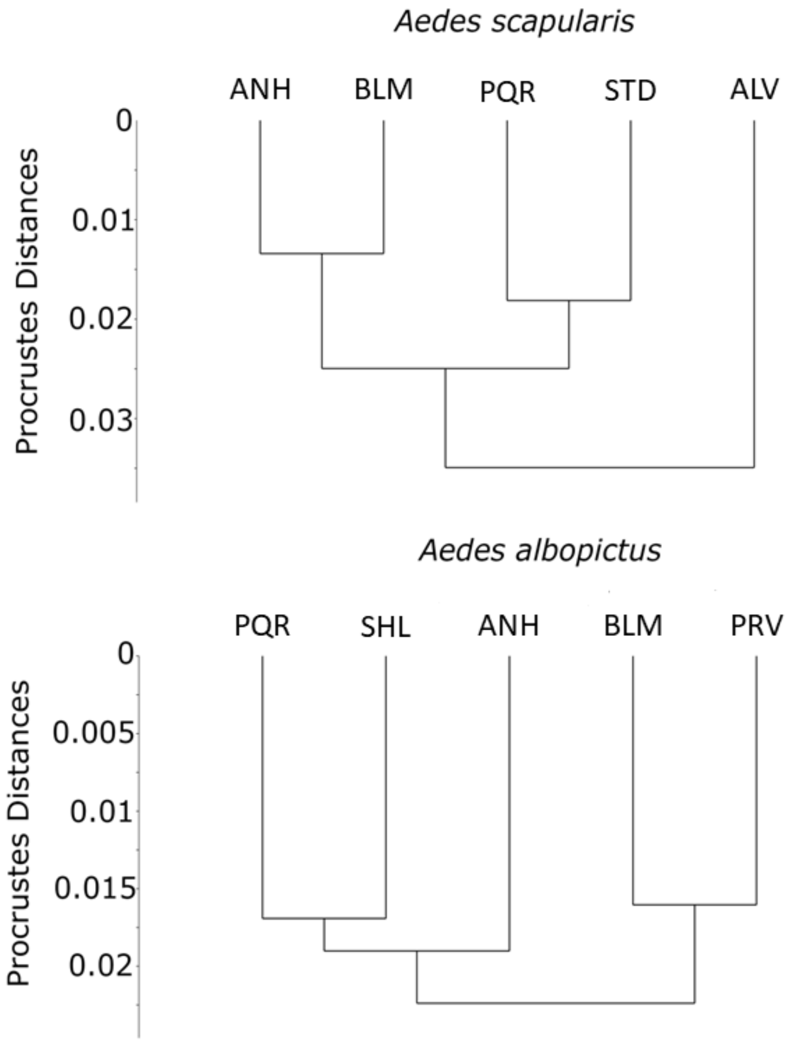

Figure S1. UPGMA phenogram of *Aedes scapularis* and *Aedes albopictus* based on Procrustes distances.
